# The role of monocytosis and neutrophilia in atherosclerosis

**DOI:** 10.1111/jcmm.13462

**Published:** 2018-01-24

**Authors:** Dimitry A. Chistiakov, Andrey V. Grechko, Veronika A. Myasoedova, Alexandra A. Melnichenko, Alexander N. Orekhov

**Affiliations:** ^1^ Department of Neurochemistry Division of Basic and Applied Neurobiology Serbsky Federal Medical Research Center of Psychiatry and Narcology Moscow Russia; ^2^ Federal Scientific Clinical Center for Resuscitation and Rehabilitation Moscow Russia; ^3^ Skolkovo Innovative Center Institute for Atherosclerosis Research Moscow Russia; ^4^ Laboratory of Angiopathology Institute of General Pathology and Pathophysiology Russian Academy of Sciences Moscow Russia

**Keywords:** atherosclerosis, monocyte, neutrophil, chemokines, inflammation, atherosclerotic plaque

## Abstract

Monocytosis and neutrophilia are frequent events in atherosclerosis. These phenomena arise from the increased proliferation of hematopoietic stem and multipotential progenitor cells (HSPCs) and HSPC mobilization from the bone marrow to other immune organs and circulation. High cholesterol and inflammatory signals promote HSPC proliferation and preferential differentiation to the myeloid precursors (*i.e*., myelopoiesis) that than give rise to pro‐inflammatory immune cells. These cells accumulate in the plaques thereby enhancing vascular inflammation and contributing to further lesion progression. Studies in animal models of atherosclerosis showed that manipulation with HSPC proliferation and differentiation through the activation of LXR‐dependent mechanisms and restoration of cholesterol efflux may have a significant therapeutic potential.

**Table:** 

**• Introduction**
**• High‐cholesterol levels increase chemokine signalling**
**• Role of different monocyte subsets in atherosclerosis**
**• NR4A1‐deficient animal model**
**• Factors that associate impaired fat metabolism with the inflammatory responses in atherosclerosis**
‐ Liver X receptors
‐ High cholesterol and hematopoietic progenitor cells
**• The repertoire of hematopoietic progenitor cells is biased towards the preferential myeloid production in atherogenesis**
**• Mechanisms of monocyte release from the bone marrow**
**• Local macrophage proliferation is a major source of lesional macrophages**
**• Contribution of monocytes to plaque progression**
**• Factors that regulate macrophage retention or emigration from the plaque**
**• Neutrophils and their role in atherosclerosis**
‐ Function and characteristics of neutrophils
‐ Components of neutrophil granules
‐ Neutrophilia and hypercholesterolaemia
‐ Mediators of neutrophil recruitment to lesions
‐Neutrophil granule components in atherosclerosis
‐ NETs in atherosclerosis
**• Conclusion**
**• Acknowledgement**
**• Conflict of interest**

## Introduction

Atherosclerotic disease is a chronic process associated with the arterial inflammation, accumulation of lipids in the vessel wall, plaque formation, thrombosis and late mortal complications such as myocardial infarction and ischaemic stroke 1. High‐blood cholesterol content and increased level of low‐density lipoprotein (LDL) were shown to primarily contribute to atherogenesis both in humans and in animal experimental models 2. During atherogenesis, cholesterol, oxidized LDL (oxLDL) and other lipids accumulate in the vascular wall contributing to the formation of the lipid core and then necrotic core of the plaque. Treatment with statins, which is focused on lowering serum lipid levels results in significant reduction of circulating lipid levels and, in general, in a one‐third decrease in the incidence of acute cardiovascular events 3. Furthermore, experimental animals, such as rodents, have innately low‐LDL content in the blood (about 50 mg/dl or less) 4 and are invulnerable to atherogenesis 5. Together, these observations indicate hypercholesterolaemia as an independent risk factor for atherosclerotic disease.

In addition to lipid deposits, the lipid core of the lesion also contains multiple types of infiltrated leucocytes. There are neutrophils, T cells, mast cells and dendritic cells 6, 7, 8. However, the majority of intraplaque immune cells are monocytes and macrophages that are derived from them. The monocytes exhibit heterogeneity: the most frequent subset (up to 90%) in humans is CD16^−^CD14^+^, while in mice there are Ly6C^high^ cells termed as classical monocytes that are differentiated to pro‐inflammatory macrophages during inflammation. The minor monocyte population (called patrol, resident or non‐classical monocytes) is represented by the CD16^+^CD14^dim^ subset in people and by the Ly6C^low^ cells in mice 9. Of these two subpopulations, the classical monocytes tend to accumulate in the plaques and give rise to pro‐inflammatory (M1) macrophages 10. These findings therefore underline the key role of both inflammation and dyslipidaemia in the atherogenic process 11, 12.

Monocytosis is an elevation of monocyte counts circulating in the blood. Commonly, monocytosis occurs in acute and chronic inflammatory conditions including atherosclerosis. Early epidemiological studies showed that increased numbers of leucocytes in the circulation can indicate higher risk of cardiovascular disease 13. Among leucocytes, further studies underlined the association of elevated levels of monocytes and neutrophils with atherosclerotic progression 14, 15, 16. In experimental murine models of atherogenesis, numbers of blood neutrophils and Ly6C^high^ monocytes were shown to correlate with atherosclerotic progression 10, 17, 18, 19. In humans, a direct correlation between the high‐cholesterol levels and increased counts of peripheral mononuclear phagocytes was also found therefore reflecting the stimulating contribution of lipids on the levels of monocytes and neutrophils in the circulation 20. Treatment with lipid‐lowering agents caused decrease in levels of both neutrophils and monocytes 10, 21 thereby suggesting for the positive link between hypercholesterolaemia and pro‐atherogenic monocytosis/neutrophilia.

In this review, we discuss the mechanisms by which hypercholesterolaemia is associated with the atherogenic process.

## High‐cholesterol levels increase chemokine signalling

Increase in the numbers of blood neutrophils may arise from the mechanisms that stimulate departure of neutrophils from the bone marrow and weaken their tissue homing 18. Neutrophils use the CXC chemokine receptor 2 (CXCR2)‐dependent mechanism [or interleukin‐8 (IL‐8)‐dependent in people] and their ligands to go out from the bone marrow and accumulate to the plaque 18, 22. Increased levels of cholesterol up‐regulate CXCR2 expression in neutrophils thereby promoting their exit from the bone marrow 23. In LDL receptor (LDLR)‐deficient mice (a hypercholesterolaemic model that spontaneously develops atherosclerosis when fed on a fat‐rich diet), knockout of CXCR2 resulted in attenuated atherogenesis indicating the pro‐atherosclerotic role of increased expression of this receptor, especially in neutrophils 24. For senescent neutrophils, the CXCR4/CXCL12 pathway serves as a mechanism to mediate the retrieval of these cell to the bone marrow 25. CXCR4‐inhibiting agents and lipid‐enriched feeding down‐regulate CXCL12 production in the bone marrow and thus led to increased levels of neutrophils and enhanced atherogenesis 18, 26.

Monocytes leaving the bone marrow and recruitment to the lesion are regulated by the C‐C chemokine receptor type 2 (CCR2) 27, 28, 29. In CCR2‐deficient mice, atherosclerosis progression is delayed due to reduced recruitment of monocytes to atherosclerotic lesions 17, 30. In people, increased plasma cholesterol is associated with higher levels of CCL2 that binds to CCR2 23. These findings can suggest that CCR2/CCL2 mechanism plays a role in the mobilization of monocytes to be pro‐atherogenic. Additionally, increased progression to monocytes and neutrophils was detected in the bone marrow in individuals affected by cardiovascular disease.

Other chemokine receptors such as CCR5 and CX3CR1 are also important to the recruitment of monocytes to atherosclerotic plaques as ablation of these receptors and CCL2 prevents monocytosis and dramatically reduces atherosclerosis in apoE‐deficient mice 17. Various monocyte subsets differentially use CCR2, CX3CR1 and CCR5 to accumulate in atherosclerotic lesions. In mice, there are two main subsets producing different chemokine receptor patterns: CCR2^+^CX3CR1^+^Ly6C^high^ and CCR2^−^CX3CR1^++^Ly6C^low^ monocytes. To enter lesions, CCR2^−^CX3CR1^++^Ly6C^low^ monocytes do not use CX3CR1 but are partially dependent on CCR5. In contrast, CCR2^+^CX3CR1^+^Ly6C^high^ monocytes employ CCR2, and CCR5 27.

Recently, Quintar with co‐authors employed a new method, long‐term 2‐photon intravital microscopy, to assess the behaviour and frequency of patrolling CX3CR1^high^Ly6C^low^ monocytes in live CX3CR1^+^/^GFP^(green fluorescent protein) mice 31. The numbers of patrolling monocytes became increased by ninefold in CX3CR1‐GFP (green fluorescent protein) mice and by 22‐fold in CX3CR1^+/GFP^/apoE‐deficient mice fed on cholesterol‐rich diet. In atherosclerosis, the real attachment of patrolling monocytes to the endothelium was observed. These data indicate that patrolling monocytes can be recruited to the atherosclerotic endothelium in a CX3CR1‐independent manner 31. In summary, CCR2, CX3CR1 and CCR5 play independent and additive roles in atherogenesis. Signals mediated *via* these pathways determine the frequency of monocyte subsets and therefore account for almost all macrophage accumulation into atherosclerotic vessels.

## Role of different monocyte subsets in atherosclerosis

Monocyte population is heterogeneous and consists of three subpopulations characterized by the differential expression of the lipopolysaccharide (LPS) receptor (CD14) and the FcγIII receptor (CD16). There are classical CD14^++^CD16^−^, intermediate CD14^++^CD16^+^ and non‐classical CD14^+^CD16^++^ monocytes in humans 32. In non‐inflammatory conditions, classical CD14^++^CD16^−^ cells are prevalent accounting for up to 90% of all monocytes while the subset of monocytes that highly express CD16 is minor (5–10% of a total monocyte population) 33. However, in inflammation, counts of CD14^+^CD16^++^ monocytes rapidly increase 34. These cells show macrophage‐like behaviour because they have the ability to phagocytosis and reactive oxygen species (ROS) production. Compared to classical monocytes, the ability of CD14^+^CD16^++^ cells to produce cytokines is greatly decreased 35. Additionally, CD14^+^CD16^++^ monocytes express intracellular adhesion molecule‐1 (ICAM‐1), opsonin receptors FcγRII and FcγRI, and high levels of major histocompatibility (MHC) class II molecules 36. Classification of the heterogeneous population of monocytes is the subject of numerous studies and is reviewed elsewhere 37.

Epidemiological and cross‐sectional studies showed association of counts of CD16‐positive (both intermediate and non‐classical) monocytes with various cardiovascular risk characteristics. For example, Schlitt *et al*. 38 reported positive correlation of increased amounts of CD14^+^CD16^+^ cells with coronary atherosclerosis onset and higher serum levels of tumour necrosis factor (TNF)‐α. In patients with unstable angina pectoris, it was found that elevated counts of CD14^+^CD16^+^ monocytes positively correlate with coronary fibrous cap thickness 39. In patients with stable angina pectoris, association between CD14^+^CD16^+^ monocyte frequencies and advanced vulnerability characteristics of atherosclerotic lesions was also shown 40. Timmerman with co‐authors found that subjects with low‐physical activity had higher levels of inflammatory CD16‐positive monocytes than physically active people 41. In low‐risk individuals, a relationship between CD16‐positive monocytes and both obesity and subclinical atherosclerosis was reported 42.

However, these early studies considered association of a whole population of CD16‐positive monocytes with cardiovascular risk factors but did not discriminate between the impact of CD14^++^CD16^+^ and CD14^+^CD16^++^ subsets. Finding of the heterogeneity of the population of CD16‐positive monocytes initiated studies aimed to evaluate a distinct role of each subset. Poitou with co‐authors described a major impact of fat mass variations on CD14^+^D16^+^ monocyte subset 43. Similarly, an intermediate monocyte subset was found to be an independent predictor of adverse cardiovascular events and sudden death in patients with chronic kidney disease (CKD) 44, 45. The role of elevated CD14^++^CD16^+^ cell numbers as a prognostic marker of unfavourable cardiovascular events was also shown in a randomly selected Swedish population 46 and in a cohort study of at‐risk German patients referred for coronary angioplasty 47.

Thus, these findings show that increased amounts of CD16‐positive monocytes and especially the CD14^++^CD16^+^ subset are associated with cardiovascular disease and may promote plaque progression and instability. In CKD patients, a greater adhesion ability of CD14^+^CD16^+^ monocytes to endothelial cells was observed, because of enhanced expression of chemokines that promote adhesion 48. Accordingly, in *in vitro* cell model of prolonged lifespan of healthy monocytes, it was found that senescent CD14^+^CD16^+^ monocytes possess enhanced ability interact with endothelial cells, increased antigen‐presenting capacity, and higher expression of inflammatory cytokines compared with the CD14^++^CD16^−^ subset 49. These observations at least in part can explain association of CD14^+^CD16^+^ monocytes with increased cardiovascular risk as such pro‐inflammatory monocytes have advanced properties for transendothelial trafficking to the intima media where they can initiate inflammation.

## NR4A1‐deficient animal model

Nuclear receptor NR4A1 (also known as Nur77) is a transcriptional factor, that is crucially involved in the development of monocytes especially patrolling Ly6C^−^ monocytes 50. Like in humans, there are three subsets of murine monocytes characterized by high (Ly6C^high^ monocytes), intermediate (Ly6C^middle^ monocytes) and low (Ly6C^low^ monocytes) of Ly6C, an inflammatory monocyte marker. The Ly6C^high^ subset exerts pro‐inflammatory properties and phagocytic activity, Ly6C^high^ cells are also pro‐inflammatory, while Ly6C^low^ monocytes have patrolling function and are involved in tissue repair 51.

NR4A1 deficiency was shown to aggravate atherosclerosis progression both in ApoE‐ 52 and LDLR‐deficient mice 53 fed on high‐fat diet. This is accompanied with dramatic reduction of Ly6C^low^ monocyte counts 53. Furthermore, in NR4A1/LDLR‐deficient mice, macrophages were polarized towards the pro‐inflammatory M1 phenotype characterized by increased expression of pro‐inflammatory markers such as toll‐like receptor 4 (TLR‐4), TNF‐α, inducible NO‐synthase (iNOS), and nuclear factor κB (NF‐κB), decreased expression of Arginase‐1 and enhanced lipid accumulation 53. Thus, these data suggest for the anti‐atherogenic role and anti‐inflammatory role of NR4A1 and Ly6C^−^ monocytes. Loss of protective Ly6C^low^ monocytes in NR4A1‐deficient mice may therefore be associated with increased macrophage activation and phenotypic pro‐inflammatory M1 polarization of macrophages and lead to advanced atherogenic progression. Ly6C^low^ monocytes dampen inflammation by releasing IL‐10, an anti‐inflammatory cytokine and differentiate to M2 macrophages after extravasation to the tissue 51. Patrolling Ly6C^low^ monocytes possess a vasculoprotective function as depletion of this population in NR4A1/apoE‐deficient mice was shown to lead to massive apoptosis of arterial endothelial cells 31.

## Factors that associate impaired fat metabolism with the inflammatory responses in atherosclerosis

### Liver X receptors

In myeloid cells, especially in mononuclear phagocytes, liver X receptors (LXR‐α and LXR‐β) play the main role in the regulation of the cholesterol reverse transport from monocytes/macrophages, thereby contributing to the lipid efflux and to the liberation of tissues from the ectopic fat deposition 54. LXRs can be activated by oxysterols and by‐products of their biosynthesis 55. The physiological goal of the activation of LXRs is the counteracting to the cholesterol excessive influx that comes from the intestine, liver and bile in order to promote its excretion 56. In addition, LXRs are involved in the immune control, such as down‐regulation of expression of pro‐inflammatory genes including TNF‐α, TLR‐4 and IL‐1β 57, 58, 59.

LXR‐dependent pathways connect the lipid metabolism with inflammation. Both LXR‐α and LXR‐β can be activated by oxysterols and cholesterol biosynthesis metabolites 60. LXRs can suppress inflammation by inhibiting NF‐kB‐dependent signalling pathways *via* the mechanism of repression 61. LXRs are also involved to the induction of anti‐inflammatory mechanisms in monocytes/macrophages by the regulation of the phagocytosis of apoptotic cells and through up‐regulating expression of arginase that represent an M2‐specific marker 62. The anti‐inflammatory properties of macrophage‐expressed LXRs were shown by the engraftment of the bone marrow from the LXR‐α‐ and LXR‐β‐deficient macrophages to ApoE‐ and LDLR‐deficient mice has already been shown 63.

LXR agonists, such as GW3965, were found to demonstrate anti‐atherosclerotic properties by inducing the plaque regression and influencing lesional composition through decrease of the content of inflammatory cells and the atheroma volume 64, 65, 66. The inhibitory effect of LXR up‐regulation was shown on plaque progression, even when there were no changes in plasma lipid profile. It is likely that LXRs can stimulate the atheroprotective trafficking of lipids from the tissues back to the liver thereby decreasing their accumulation in peripheral organs/tissues 67.

In addition, LXRs participate in the clearance of senescent neutrophils to maintain the neutrophil homoeostasis and prevent excessive amplification of neutrophils. In mice, LXR deficiency is associated with perturbations in the neutrophil homoeostasis that leads to enhanced granulopoiesis 68. LXR mediate the phagocytosis of aged neutrophils through the activation of the proto‐oncogene tyrosine‐protein kinase MER (MERTK) that leads to enhanced phagocytosis by macrophages 69. Treatment with GW3965 and TO901317 (both are agonists of LXRs) resulted in reduced counts of neutrophils in the circulation 70. In addition, in LDLR‐deficient mice, Kappus *et al*. 71 showed the ability of both LXR agonists to down‐regulate expression of inflammatory genes in macrophages lacking ABCA1/G1. This in turn suggests for existence of anti‐inflammatory signalling pathways that are independent from the mechanism of cholesterol efflux.

### High cholesterol and hematopoietic progenitor cells

Hematopoietic stem and multipotential progenitor cells (HSPCs) born in the bone marrow is crucially involved in the production of monocytes and neutrophils. In physiological conditions, neutrophils and monocytes are directly originated from HSPCs that are located in special microenvironments of the bone marrow called stem cell niches. Normally, the production and mobilization of HSPCs are strictly controlled to avoid excessive formation of monocytes/macrophages but to maintain the permanent self‐renewal of these cell populations during the life 72.

In inflammation, these progenitors were shown to be primarily involved in the mobilization of pro‐inflammatory cells 73. Cholesterol metabolism regulates the function of HSPCs and their ability to differentiate to monocytes and other immune cells. In animal models of atherosclerosis, a sufficient extramedullary hematopoiesis was observed. In mice, HSPCs can be mobilized from the bone marrow to the secondary lymphoid organs such as spleen where they can differentiate to monocytes and be involved in atherosclerosis through the recruitment to the plaque 74. Normally, the spleen serves as a source for monocytes but can significantly increase the production of these cells in inflammatory and hypercholesterolaemic conditions 75. Cholesterol‐rich diet permanently increases counts of splenic monocytes indicating the positive role of high‐cholesterol levels on the mobilization of HSPCs from the bone marrow 76.

**Figure 1:**
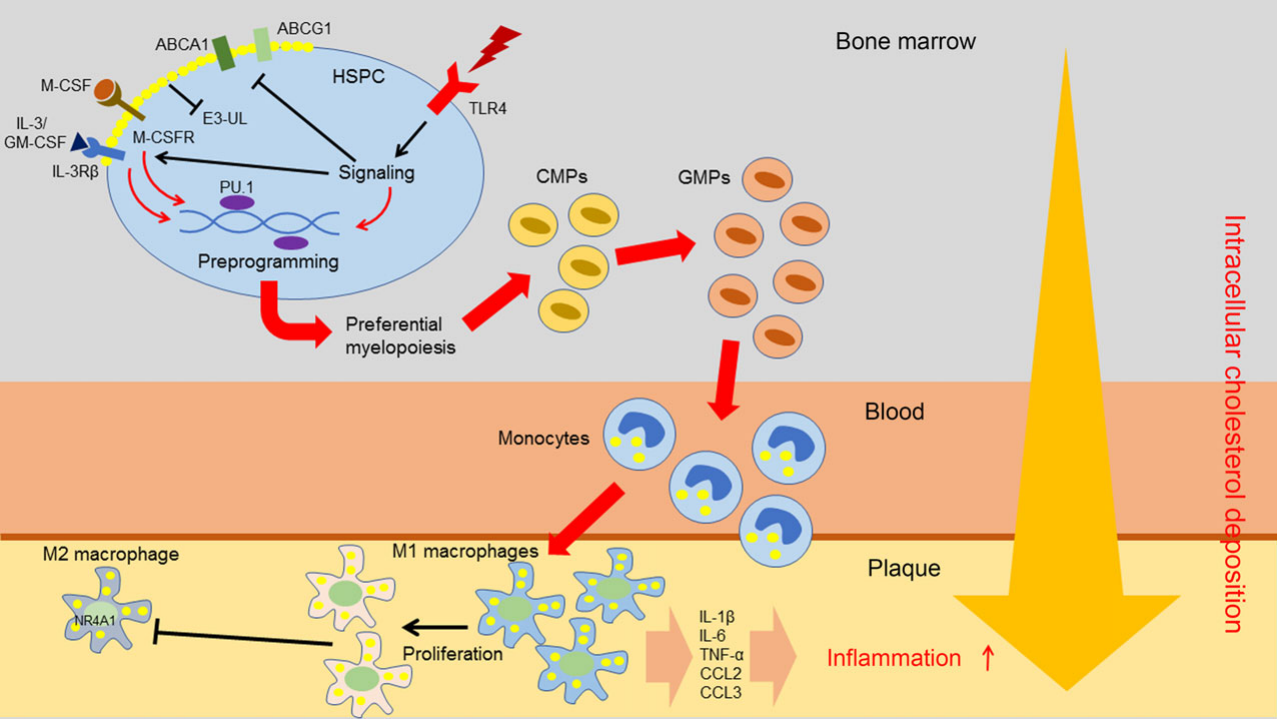
Contribution of high cholesterol‐induced myelocytosis to atherogenesis. High cholesterol up‐regulates expression of inflammatory receptors such as TLR‐4 on the surface of HSPCs. TLR‐4 can be activated by pro‐inflammatory stimuli. TLR‐4‐dependent signalling leads to the inhibition of cholesterol efflux mediated by ABCA1 and ABCG1. Increased membrane cholesterol leads to increased surface expression of myeloid cytokine receptors IL‐3Rβ and M‐CSFR due to failure to activate E3‐ubiquitin ligases (E3‐UL). The receptors mediate enhanced signalling from myeloid cytokines (IL‐3, M‐CSF, GM‐CSF), which in cooperation with cholesterol‐enriched microenvironment promote HSPC reprogramming (mediated by transcription factor PU.1) to produce more myeloid cells. During differentiation to common myeloid progenitors (CMPs) and further to granulocyte‐macrophage progenitors (GMPs), myeloid cells uptake more cholesterol since their reverse cholesterol transport is suppressed. In the circulation, monocytes can accumulate more cholesterol before entering the plaque. In the plaque, lipid‐laden monocytes preferentially differentiate to M1 macrophages that produce a number of pro‐inflammatory cytokines and therefore contribute to inflammation. In these macrophages, expression of transcription factor NR4A1 is suppressed thereby limiting the ability to switch to the anti‐inflammatory M2 phenotype.

Moreover, high cholesterol can also induce ‘memory’ effects (through presumably the epigenetic changes) in HSPCs on their direct progeny (Fig. 1). For example, Seijkens with co‐authors reported enhanced ability of bone marrow‐derived HSPCs transplanted from LDLR‐deficient mice to normal mice to induce generation of myeloid cells particularly granulocytes and Ly6C^high^ monocytes 77. High cholesterol‐induced HSPCs also induce pro‐inflammatory and pro‐atherosclerotic M1 macrophages that can subsequently produce inflammatory messengers, such as TNF‐α, IL‐6 and CCL2. In addition, enhanced properties of hypercholesterolaemia‐induced HSPCs were shown to then differentiate to pro‐inflammatory monocytes that can accumulate in the plaque and promote the atherosclerotic development 77, 78. However, it should be mentioned that pro‐inflammatory effects of high cholesterol‐induced HSPCs may be mediated not only through epigenetic reprogramming but also through up‐regulation of the IL‐3/macrophage colony‐stimulating factor (GM‐CSF) receptor 79.

Recently, van Kampen with co‐authors showed the ability of the bone marrow‐derived HSPCs from the normal recipient mice to which hypercholesterolaemia‐primed HSPCs were engrafted to induce hypomethylation of CpG regions in the PU.1 and interferon regulatory factor 8 (IRF‐8) genes 80. Both PU.1 and IRF‐8 are transcription factors that control commitment of the myeloid lineage 81. In HSPCs, high cholesterol‐induced epigenetic changes in the promoters of the PU.1 and IRF‐8 genes lead to the transcriptional de‐repression of both genes and their enhanced expression that in turn primes preferential differentiation of HSPCs to the myeloid cells and atherogenesis (Fig. 1) 77. Hematopoietic IRF‐8‐deficiency promotes atherosclerosis in mice 19. Similar with hypercholesterolaemia, hyperglycaemia was shown to up‐regulate myelopoiesis and monocytosis in diabetic mice 82.

ATP‐binding cassette (ABC) transporters such as ABCA1 and ABCG1 are involved to the reverse transport thereby preventing its excessive accumulation in the cell. In LDLR‐deficient mice with genetic deletion of both ABCA1 and ABCG1, increased numbers of leucocytes and enhanced atherosclerosis was observed 83. The mechanism by which ABCA1/ABCG1 deficiency contributes to atherogenesis involves increased proliferation of HSPCs 84. Elevated proliferation of HSPCs correlates with enhanced formation of cholesterol‐rich lipid rafts, up‐regulation of ERK1/2 phosphorylation, RAS activation and increased expression of the IL‐3/GM‐CSF receptor 79. Treatment of ABCA1/ABCG1‐deficient HSPCs with cyclodextrin (a cyclic oligosaccharide that solubilizes cholesterol and therefore abolishes cholesterol‐rich lipid rafts) significantly decreases proliferation of HSPCs 84. Increased deposition of cholesterol in ABCA1/ABCG1‐deficient HSPCs elevates cholesterol content in cell membrane, enhances generation of lipid rafts and increases surface expression of the IL‐3/GM‐CSF receptor that shares a common CD131 subunit. IL‐3/GM‐CSF stimulate Ras/ERK‐dependent signalling, which leads to enhanced proliferation of HSPCs 84 and elevated production of monocytes and neutrophils 85.

In LDLR‐deficient mice transgenic for ApoA1 and fed on high‐fat diet, transplantation of the ABCA1/ABCG1‐deficient bone marrow resulted in significantly decreased atherogenesis compared with non‐transgenic LDLR‐deficient mice HSPCs 84. These observations suggest on the anti‐atherogenic role of ApoA1 and HDL that in part are released through the inhibition of proliferation of HSPCs and subsequent monocytosis and neutrophilia.

ApoE is another anti‐atherogenic lipoprotein that is mainly involved in the clearance of cholesterol‐containing LDL particles and vasculoprotective effects in arteries 86. ApoE was shown to be able to regulate the proliferation of HSPCs 78. Bellosta with co‐authors observed increased propensity of hypercholesterolaemic ApoE‐deficient mice fed on high‐cholesterol diet to develop increased counts of HSPCs, neutrophils and monocytes, while LDLR‐deficient or ApoA1‐deficient mice exhibited only a mild predisposition to monocytosis and neutrophilia. In humans, individuals that have low HDL levels because of genetic mutations do not show increase in numbers of circulating HSPCs and monocytes 87.

The mechanism by which ApoE can down‐regulate HSPC proliferation may involve binding of HSPC‐secreted ApoE to the surface proteoglycans and stimulation of ABCA1/ABCG1, which in turn activates increased reverse cholesterol transport from HSPCs. ABCA1/ABCG1‐deficient HSPCs are especially prone to cholesterol deposition and are sensitive to IL‐3‐ and GM‐CSF‐dependent proliferation‐stimulating effects, which in turn accelerates atherogenesis 10, 18, 87. The treatment of ApoE‐deficient mice with phospholipid/ApoA‐I complexes (that are poor of phospholipids) decreased the overproduction of HSPCs and monocytes/neutrophils 78. Additionally, administration of LXR agonists also had atheroprotective effects *via* elevating ApoE levels in HSPCs and reducing proliferation of myeloid precursors and monocytes/neutrophils 78.

In summary, cholesterol regulates HSPC proliferation through the mechanism of the reverse cholesterol transport mediated by the activity of ABCA1/ABCG1 and its interaction with ApoE, an acceptor protein for cholesterol. The strong interaction between ApoE and both cholesterol efflux pumps results in the decrease of cholesterol content in the plasma membrane and therefore weaken of IL‐3/GM‐CSF‐dependent mitogenic signals. The ApoE‐dependent proliferative mechanism is independent on HDL or ApoA1 but can be stimulated by the presence of these free cholesterol acceptors or through the activation of LXRs 78.

## The repertoire of hematopoietic progenitor cells is biased towards the preferential myeloid production in atherogenesis

Vascular inflammation that chronically persists in atherosclerosis can alter the lineage selection and differentiation of HSPCs towards a preferential generation of pro‐inflammatory cells. In hypercholesterolaemic mice and humans with familial hypercholesterolaemia, an obvious increase in monocyte production was shown along with increased expression of LDL‐handling molecules, such as LDLR, scavenger receptor B1 (SRB1) and low‐density lipoprotein receptor‐related protein 1 (LRP1) 88.

ApoE‐deficient murine macrophages were shown to develop a more pronounced response to TLR‐4 ligands compared with normal cells. In line with that, ABCA1/ABCG1‐deficient macrophages exhibit higher surface expression of TLR‐4 and more sensitive to TLR‐4‐dependent stimuli in comparison with the wild‐type macrophages 71, 89. As TLR‐4 is involved in the recognition of LPS, other bacterial and viral components, TLR‐mediated induction of the pro‐inflammatory response can mediate skewing the preferential differentiation of HSPCs to inflammatory cells observed in acute (such as an infection) and chronic (such as atherosclerosis) inflammatory environment. (Fig. 1). This pathway can exist because TLR‐4 expression was found in HSPCs 90. Furthermore, increased expression of TLR‐4 in HSPCs can cooperate with impaired cholesterol metabolism in HSPCs (in the initiation of the bias to increased generation of myeloid precursors in individuals affected by hypercholesterolaemia, atherosclerosis and other cardiometabolic disorders associated with altered lipid homoeostasis). In inflammation, some new substances, such as oxLDL 91, S100A8/A9 92 and HSP‐70 93 can activate TLR‐4 dependent responses in HSPCs and indeed induce myeloid skewing in HSPC differentiation. However, specifically to atherosclerosis, more studies are required to establish a range of TLR‐4 ligands capable to influence the HSPC differentiation in the atherosclerotic disease. In any case, TLR‐4 activation was shown to dampen the activity of ABCA1/ABCG1 transporters and therefore decrease cholesterol efflux from the cells. In addition, TLR‐4 stimulation increases expression of inflammatory cytokines and chemokines in macrophages thereby contributing to the pro‐inflammatory polarization of macrophages 94. However, it should be definitely shown whether the cooperation between TLR‐4 signalling and impaired lipid metabolism in HSPCs can contribute to the predominant differentiation of HSPCs to the myeloid lineage in atherosclerosis.

## Mechanisms of monocyte release from the bone marrow

HSPC‐derived monocytes can retain in the bone marrow and intravasate into the bloodstream in a circadian rhythm‐dependent manner 95. In steady‐state conditions, oscillatory release of monocytes from the blood is controlled by oscillating expression of circadian genes Bmal1, Nrld1 and Dbp in monocytes. For example, the clock gene Bmal1 regulates egress of Ly6C^high^ monocytes through controlling expression of chemokines Ccl2 and Ccl8 in monocytes 95. When Bmal1 reaches a nadir at the transition to the active phase, Bmal1‐mediated repression of Ccl2 disappears. This allows Ccl2 levels to rise to peak levels and hence promotes monocyte mobilization to the bloodstream and inflamed peripheral tissues.

In pathological conditions, monocyte emigration from the bone marrow can be promoted by infection or sterile inflammation such as myocardial infarction (MI) and atherosclerosis. Shi *et al*. 29 showed that challenge with LPS leads to the departure of inflammatory Ly6C^high^CCR2^+^ monocytes from the blood marrow. The departure rate is highly correlated with expression of CCL2, a ligand for CCR2, by mesenchymal stem cells and reticular cells presented in the bone marrow. Accordingly, depletion of CCL2 in these cells abolishes leaving of Ly6C^high^CCR2^+^ monocytes from the bone marrow 29.

In myocardial infarction‐induced heart injury, leucocyte‐expressed β_2_‐adrenergic receptor (ADRB2)‐dependent signalling was shown to be important for the recruitment of monocytes/macrophages and neutrophils to the damaged heart for induction of cardiac remodelling 96. In Adrb2‐deficient bone marrow, CCR2 expression and responsiveness to CCL2‐mediated migration were abolished. In contrast, ADRB2 activation leads to the up‐regulation of CCR2 expression and migratory responsiveness to CCL2 in the bone marrow. Mechanistically, β‐arrestin2‐biased ADRB2 signalling is required for the regulation of CCR2 expression. Additionally, activator protein 1 (AP‐1), a transcriptional factor, is needed in mediating CCR2 expression in response to ADRB2 stimulation in both murine bone marrow and human monocytes 97. Therefore, ADRB2 expression in immune cells is essential for the initiation of early inflammatory repair response to acute cardiac damage by assisting in infiltration of the infarct by leucocytes.

## Local macrophage proliferation is a major source of lesional macrophages

During plaque growth, macrophages progressively accumulate in the diseased arterial wall. Previous concept considered monocyte infiltration as a major source that gives rise to intraplaque macrophages. However, Robbins *et al*. 98 showed that population of macrophages in advanced plaques of apoE‐deficient mice is predominantly replenished through the mechanism of local macrophage proliferation rather than through macrophage influx. So far, mechanisms that drive local macrophage proliferation are not well understood. GM‐CSF, a primary candidate to serve as a mitogenic signal, is not responsible for macrophage proliferation as this growth factor was shown to mediate proliferation of dendritic cells in the hypercholesterolaemic aortic intima 99. Scavenger receptor A1 (SRA1) abundantly presented on the surface of macrophages was suggested as a key player in macrophage proliferation 100. SRA1 transfers oxLDL into the cell and therefore can contribute to the transformation of macrophages to foam cells in the local lipid‐rich and pro‐inflammatory microenvironment 101. Recent studies showed that hypercholesterolaemia and improper reverse cholesterol transport can cause enhanced proliferation and mobilization of bone marrow‐derived HSPCs, excessive proliferation of splenic myeloid progenitors and monocytes, and increased monocyte influx to the plaque 78, 84, 102. All these cell types can be involved in monocytosis and support enhanced monocyte influx to the plaque. Indeed, intraplaque hypercholesterolaemic microenvironment may represent a key force that drives local amplification of macrophages. However, this possibility should be elucidated.

## Contribution of monocytes to plaque progression

Plaque evolution is a complex multistep process. Plaque formation and progression include many consequent events and mechanisms including intimal lipoprotein retention, recruitment of inflammatory cells, foam cell generation and death, VSMC proliferation and migration, extracellular matrix (ECM) synthesis, neovascularization, calcification, arterial wall remodelling, fibrous cup rupture, thrombus formation and other events. Pathogenic mechanisms of atherogenic progression are comprehensively characterized in several recent reviews 103, 104, 105.

Monocytes play an important role in atherogenesis. In the plaque, inflammatory monocytes represent a significant portion of immune cells. Inflammatory monocytes release inflammatory messengers such as TNF‐α, IL‐1β and CCL2 and produce ROS therefore propagating vascular inflammation and promoting oxidative stress 106. In addition to the ability to produce cytokines, mouse inflammatory Ly6C^+^ monocytes can extravasate through the endothelial barrier to the subendothelial space. The transendothelial trafficking is mediated by interaction of complementary pair CCR2/CCL2 or/and CCR5/CCL5 in a VLA1/VCAM‐1 dependent manner 51. In the arterial intima, Ly6C^+^ monocytes differentiate to inflammatory and pro‐atherogenic M1 macrophages that actively promote plaque formation and progression through generation of foam cells and release of inflammatory cytokines (TNF‐α, IL‐6), ROS and matrix metalloproteinases (MMPs). By degrading ECM, macrophage‐derived MMPs contribute to the fibrous cup rupture, intraplaque neovascularization and pathological vascular remodelling 107. M1 macrophages express MHC‐I/II molecules and therefore can present antigens to CD4^+^T cells and initiate an adaptive immune response 108.

Like M1 macrophages, inflammatory monocytes can also present antigens to CD4^+^ T cells in a CD40‐dependent fashion. CD40/CD40L signalling pair plays a central role in inducing adaptive immunity in atherosclerosis 109. CD40 is expressed on the surface of dendritic cells, macrophages and monocytes, while CD40 ligand (CD40L) is produced by CD4^+^T cells and platelets. Both CD40 and its ligand are expressed by vascular smooth muscle cells (VSMCs) and endothelial cells 110. In atherosclerosis, oxLDL was suggested as a potential self‐antigen for the activation of CD4^+^ T cells 111. In human plaques, colocalization of CD40‐CD40L with oxLDL epitopes, SRA1 and CD16 was found thereby indicating a potential antigenic role of oxLDL 112. In apoE‐deficient mice, deletion of CD40 or CD40L leads to attenuation of atherosclerosis, increasing ECM and preferential polarization towards the anti‐inflammatory M2 phenotype 113.

Human CD14^++^CD16^+^ monocytes, which are orthologs of murine Ly6C^+^ monocytes, are involved in the phagocytosis of LDL particles and apoptotic cells. CD14^+^CD16^+^ cells (both non‐classical and intermediate) perform oxLDL uptake at activated endothelial cell surfaces as they are more resistant to oxLDL‐induced lipotoxicity 114. Kapinsky with co‐authors showed that the inflammatory CD14^++^CD16^+^ subset is more prone to foam cell formation when exposed to enzymatically degraded LDL 115. This may indicate a potential pro‐atherogenic role of this subset. Overall, in atherosclerosis, monocytes mainly serve as a source of macrophages and dendritic cells but are also involved in inflammation, foam cell formation, and activation of T cells.

## Factors that regulate macrophage retention or emigration from the plaque

Lesional macrophage counts are dynamically determined by entry of circulating monocytes, and macrophage egress, local proliferation and death 116. Macrophage egress was described in early lesions. However, the emigration rate was shown to decline with plaque progression 117. Macrophage emigration stabilizes plaque phenotype and is associated with atherosclerosis regression.

The balance between retention and emigration signals is likely to be responsible for the content of intraplaque macrophages. These signals are widely unknown but become to be discovered. For example, cholesterol‐induced expression of semaphorin 3E and netrin 1 inhibits migration and stimulates retention of macrophages in the plaque 118, 119. Hypoxia also up‐regulates expression of these molecules 120, 121 and therefore contributes to macrophage retention. LDLR‐deficient mice whose bone marrow lacks netrin 1 develop less advanced atherosclerosis and exhibit enhanced macrophage egress from plaques thereby indicating that netrin 1 is involved in retention of macrophages in lesions 118. In the plaque, netrin 1 is expressed by VSMCs while macrophages express netrin receptor UNC5B. Interaction between these factors underlines molecular mechanism of macrophage retention 122.

Expression of semaphorin 3E and its receptor plexin D1 is up‐regulated in atherosclerotic lesions especially in advanced plaques. Semaphorin 3E is highly expressed by inflammatory M1 macrophages or in macrophages exposed to hypoxia or oxLDL. In a mouse model of atherosclerosis regression, expression of semaphorin 3E is greatly suppressed and associated with reduced content of macrophages in the plaque. Mechanistically, semaphorin 3E inhibits actin polymerization and macrophage motility thereby promoting immobilization of these cells in the plaque 120.

Other molecules, which suppress cell motility (*e.g*., adhesion molecules) or resolution of inflammation, may also be involved in the regulation of macrophage retention or emigration. Recently, it was reported that the adhesion receptor CD146 regulates the formation of macrophage foam cells and their retention within the lesion 123. Expression of CD146 in macrophages is up‐regulated by oxLDL. This receptor promotes internalization of the scavenger receptor CD36 involved in oxLDL uptake. This leads to increased lipid accumulation in macrophages and reduced migratory ability towards chemokines CCL19 and CCL21 123.

The signals, which stimulate macrophages to leave lesions, remain poorly defined. It seems that the macrophage fate to retain or exit a plaque is determined by a complex pattern of expression of factors that inhibit or promote motility. Further studies are required to define other such factors in animal models of regression.

## Neutrophils and their role in atherosclerosis

Neutrophils are crucially involved in atherogenesis. These cells play a sharply pro‐inflammatory role and can seriously damage arterial wall due to the multiple cytotoxic and proteolytic molecules that they carry. In atherosclerosis, inflammatory and metabolic stimuli can significantly increase neutrophil production (*i.e*., cause neutrophilia) that may have deleterious sequelae for cardiovascular system.

### Function and characteristics of neutrophils

Neutrophils belong to the family of polymorphonuclear leucocytes as they have a nucleus divided into several segments. Among leucocytes, these cells are the most frequent (up to 75% of a total population) 124. The lifespan of these cells is relatively short (8–12 hrs in the bloodstream) because neutrophils contain granules with highly toxic compounds released upon activation 125. This is a reason why they are so highly produced by the bone marrow accounting for 55–65% of the hematopoietic potential 126.

In physiological conditions, the primary task of neutrophils is to neutralize and kill various pathogens such as bacteria, fungi and protozoans. Neutrophils possess the capacity to phagocytize bacteria that then are killed by proteases and antimicrobial factors released from multiple cytoplasmic granules to the phagocytosome. In addition, stimulated neutrophils massively release ROS to the phagocytosome that damage phagocytized microorganisms 127. However, the defensive role of neutrophils is more complex because these cells were shown to interact with other leucocytes such natural killer cells, macrophages, dendritic cells and T‐ and B‐lymphocytes 128.

Neutrophils have specific advanced tools such as neutrophil extracellular traps (NETs) to kill pathogens. NETs represent a network of fibres consisted of decondensed chromatin associated with granular protein components 129. Neutrophils throw out NETs in response to pro‐inflammatory stimuli such as LPS, IL‐8. This process is accompanied with the plasma membrane destruction 130. In the NETs, chromatin creates a viscous matrix capable to capture pathogens that will be then eliminated by high concentrations of NET‐associated proteases and antimicrobial peptides [human neutrophil peptide 3 (HNP3), cathelicidin, α‐defensins] 131. NETs generation usually leads to the neutrophil death, but some cells remain mobility and ability to destroy microbes *via* phagocytosis and degranulation 132. As macrophages cannot neutralize NETs due to their high‐cytotoxic potential, such networks are destructed by exonucleases 130.

Another specific characteristic of neutrophils is a respiratory burst. Neutrophil granules contain three proteins [myeloperoxidase (MPO), NADPH oxidase and lactoferrin] involved in redox reactions and capable to produce ROS. NADPH oxidase is a significant contributor to superoxide anion radical (O2^.−^) production by activated neutrophils. This enzyme is a multicomponent assembly composed by membrane‐associated cytochrome b558 (includes subunits p22^phox^ and gp91^phox^) and three cytoplasmic subunits (p47^phox^, p67^phox^ and Rac1) 133. As NADPH oxidase activation is accompanied with increased ROS production that could damage host tissues, expression and activity of this enzyme is strictly regulated. For example, adenosine inhibits NADPH oxidase by decreasing density of cytochrome b558 components on the plasma membrane through redistribution between intracellular compartments and or/endocytosis 134. Activated neutrophils produce large amounts of the superoxide anion radical that is converted to H_2_O_2_, which becomes involved to further reactions to generate ROS 135. Massive ROS production helps to degrade bacteria and other pathogens.

Due to high reactivity and a rich arsenal of cytotoxic agents, neutrophil production and function should be strictly controlled. Neutrophils experience continuous death through apoptosis. Dead or apoptotic neutrophils are then cleared by macrophages 136. Loss of a proper control of neutrophil production or inefficient clearance may lead to neutrophilia and neutrophil overactivity associated with the development of many inflammatory, autoimmune and allergic diseases. The rate of neutrophil infiltration correlated with the severity of disease 137.

### Components of neutrophil granules

In the cytoplasm, neutrophils contain multiple granules. There are three types of granules: primary, secondary and tertiary. Primary granules contain MPO, serine proteases (cathepsin G, neutrophil elastase, proteinase 3, neutrophil serine protease 4 and azurocidin 1) and antimicrobial peptides (bactericidal/permeability‐increasing protein, defensins). MPO, cathepsin G and neutrophil elastase are the major protein components of primary granules. MPO produces hypochlorous acid (HOCl) from hydrogen peroxide (H_2_O_2_) and chloride anion (Cl^−^) during neutrophil respiratory burst. This enzyme also oxidizes tyrosine to tyrosyl radical with H_2_O_2_ 138. HOCl and tyrosyl radicals are cytotoxic agents that kill pathogenic bacteria 139. Cathepsin G is an essential component of NETs that kills microbes 140. By degrading structural ECM proteins, this protease contributes to tissue remodelling in inflamed sites 141 Capodici and Berg 1989. Neutrophil elastase contains two isoforms (2A and 2B) and degrades virulence factors of various Gram‐negative bacteria 142. This serine protease also participates in tissue remodelling by cleaving collagen‐IV and elastin 143.

Secondary granules contain several enzymes [collagenase (also known as MMP‐1), lysozyme, alkaline phosphatase, NADH oxidase), cathelicidin (antimicrobial peptide; also known as Cramp (in mice) or LL37 (in humans)] and lactoferrin, a protein with a wide spectrum of antimicrobial activity. Tertiary granules harbour gelatinases A and B (or MMP‐2 and ‐9, respectively), cathepsin (serine protease) and lipocalin 2, an antimicrobial peptide 7. Thus, neutrophil granules contain a variety of molecules with proteolytic, antimicrobial, cytotoxic and oxidative activity that are released upon degranulation and carry a potent destructive potential.

### Neutrophilia and hypercholesterolaemia

In neutrophilia, higher numbers of neutrophils circulate in the blood. Neutrophilia is usually induced as a response to bacterial infection. However, increase in neutrophils in the blood can also result from other factors. As was shown in atherosclerotic models of mouse, rabbit and swine, high cholesterol can cause rise of neutrophils in the circulation 18, 19, 144, 145. In humans, a positive correlation between neutrophil counts, rupture‐prone areas of the plaque and density of intraplaque microvessels was found in patients with carotid atherosclerosis 146. These data indicate that neutrophilia can increase lesional instability and increase risk of plaque rupture. Mechanistically, neutrophil proteases can degrade the plaque fibrous cup and contribute to destabilization of the lesion 147.

Neutrophilia was shown to serve as independent predictor for adverse cardiovascular events in subjects with asymptomatic carotid stenosis 148. Guasti with co‐authors performed systemic analysis of studies in which a prognostic value of neutrophil counts for unfavourable cardiovascular events was evaluated on a total population of 34,000 individuals with acute coronary syndromes and/or after cardiac revascularization 16. A significance of neutrophilia (if evaluated concomitantly with total leucocyte numbers and levels of C‐reactive protein) as an independent predictor for cardiovascular outcomes was shown 16.

Overall, in hypercholesterolaemic animals, neutrophilia and monocytosis can be induced concomitantly through the common mechanism involving increased surface expression of the IL‐3/GM‐CSF receptor due to cholesterol accumulation in cell membrane of HSCs. This in turn stimulates their proliferative potential 84. By studying children with familial hypercholesterolaemia, Tolani *et al*. 88 demonstrated that similar mechanism of induction of cholesterol‐induced HCS proliferation also exists in humans.

### Mediators of neutrophil recruitment to lesions

**Figure 2:**
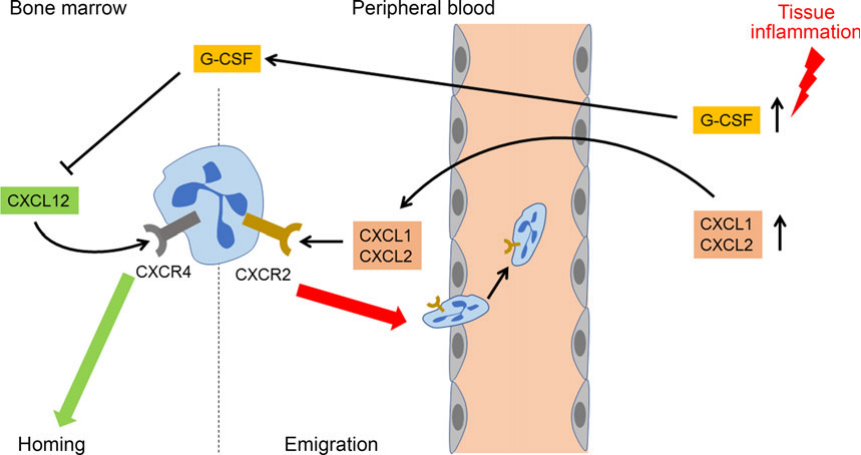
Mobilization of neutrophils from the bone marrow in inflammatory conditions. Chemokine receptor CXCR4 is responsible for retention of neutrophils in the bone marrow while CXCR2 triggers neutrophil emigration from the bone to the circulation. The CXCR4/CXCL12 axis promotes neutrophil homing in the bone marrow. In tissue inflammation, levels of G‐CSF, CXCL1 and CXCL2 are up‐regulated. G‐CSF mobilizes neutrophils from the bone marrow by increasing the ratio of CXCR4 to CXCR2 ligands in the bone marrow. Both CXCL1 and CXCL2 are CXCR2 ligands that stimulate neutrophil entrance to the bloodstream. Increased G‐CSF and CXCL1 are produced by the bone marrow and local inflamed tissue.

In the bone marrow, commitment of the neutrophil‐specific lineage is controlled by G‐CSF and IL‐23. In myeloid progenitors, down‐regulation of the reverse cholesterol transport stimulates production of neutrophils. However, neutrophils hold in the bone marrow because of the interaction of CXCL12 with its receptor CXCR4 149. Interruption of the CXCL12/CXCR4 axis or elevated plasma CXCL1/CXCL2 concentrations induces mobilization of neutrophils from the bone marrow that leads to neutrophilia (Fig. 2). CXCL1/CXCL2 bind to CXCR1 located on the surface of neutrophils and stimulates entry of these cells to the bloodstream 150. Chemotactic stimuli such as macrophage‐derived CCL3 and platelet‐derived CCL5 stimulate neutrophils *via* CCR1, CCR3 and CCR5 and promote firm attachment of neutrophils to the inflamed endothelium 151. Sensing of neutrophil‐derived CCR5 induces sialylation of this receptor mediated by the α2,3‐sialyltransferase IV (St3Gal4). This enzyme is also essential for CCL5‐mediated neutrophil recruitment and extravasation. St3Gal4‐deficient murine neutrophils exhibited decreased binding of Ccl5 and aberrant Ccl5‐mediated integrin activation. Accordingly, Ccl5‐dependent immobilization of neutrophils on the endothelial surface was almost abolished. Ccl5‐triggered neutrophil transmigration across the endothelial barrier was drastically reduced. As a result, St3Gal4/apoE‐deficient mice develop smaller and less inflammatory lesions 151.

In line with this, transplantation of the bone marrow deficient for CCL3 (a ligand for CCR1, CCR4 and CCR5) to LDLR‐deficient mice resulted in diminished aortic atherosclerosis progression likely due to prevention of intraplaque accumulation CCL3‐deficient neutrophils. Presumably, CCL3 can provide a chemotactic gradient essential for supporting neutrophil movement to the plaque 152.

CXCL1 also essential for neutrophil recruitment. In a mouse model of shear stress‐induced atherogenesis, implementation of Evasin‐3, a natural protein inhibitor of both chemokines, resulted in specific reduction of inflammation driven by neutrophils, less production of MMP‐9 and increased collagen deposits in the plaque 153. Concomitant administration of Evasin‐3 (inhibits CXC chemokines) and Evasin‐4 (inhibits CC chemokines) had beneficial effects on post‐infarction heart failure in mice by decreasing cardiac inflammation, oxidative stress and neutrophil infiltration as a result of blocking CXCL1 and CCL2 154. Similarly, in apoE‐deficient mice, treatment with a nicotinamide phosphoribosyltransferase inhibitor (FK866) diminished neutrophil and MMP‐9 content in the plaque and increased collagen content by decrease of systemic CXCL1 155.

In addition to chemokines, other proteins can regulate neutrophil recruitment. Fatty acid amide hydrolase (FAAH)/apoE‐deficient mice had smaller lesions enriched by MMP‐9 and neutrophils. Interestingly, in apoE‐deficient mice, FAAH inhibition led to increased recruitment of neutrophils but not monocytes 156. Hoyer with co‐authors observed no change in plaque size but confirmed elevated production of MMP‐9 and higher neutrophil counts in the lesions of FAAH/apoE‐deficient mice 157. FAAH catalyses degradation of anandamide, an endocannabinoid, which presumably can control mobility of leucocytes through the cannabinoid receptor type 2 (CB2R) 158. Stimulation of CB2R with a specific agonist JWH‐133 caused significant decrease neutrophil counts and MMP‐9 production in mouse aortic root plaques; a less profound reduction was observed in carotid lesions 159. In a murine model of ischaemia/reperfusion, CBR2 possessed cardioprotective functions by limiting oxidative stress and neutrophil infiltration in the infarcted myocardium 160. It was also shown that CBR2 exerts a protective function against stroke by blocking neutrophil movement to ischaemic brain regions 161. The inhibitory mechanism is consisted in CBR2‐mediated suppression of CXCL2‐dependent activation of MAP kinase p38, that is required for neutrophil migration towards CXCL2 161. Another cannabinoid receptor GPR55, which is expressed in human neutrophils, was shown to cooperate with CBR2 by suppressing neutrophil migration towards 2‐arachidonoylglycerol, a CBR2 agonist, while down‐regulating neutrophil degranulation and ROS generation 162. Thus, anti‐inflammatory effects of endocannabinoids at least in part could be explained by inhibition of neutrophil recruitment to inflamed tissues but also by blockage of neutrophil effector function 163.

Krüppel‐like factor 2 (KLF2), a transcription factor, is another regulator of neutrophil recruitment. LDLR‐deficient mice lacking KLF2 displayed increased attachment of neutrophils to endothelial cells, accumulation of neutrophils and monocytes and up‐regulation of MPO activity in aortic roots 164. KLF2‐dependent suppression of recruitment and extravasation of inflammatory cells including neutrophils appears to be attributed to its protective effects on endothelial cells by maintaining endothelial barrier integrity 165 and inhibition of expression of adhesion molecules such as E‐selectin and VCAM‐1 166. As known, KLF2 expression is regulated by mechanical forces. Steady laminar flow induces KLF2 expression while perturbed flow down‐regulates expression of this factor 167. Perturbed flow presented in atheroprone arterial regions causes pro‐inflammatory activation of the local endothelium associated with production of inflammatory messengers and adhesion molecules. This attracts inflammatory cells such monocytes and neutrophils to inflamed sites 168. Recently, Franck with co‐authors reported that disturbed bloodflow can stimulate neutrophil recruitment and accumulation, arterial denudation due to the endothelial death 169. Endothelial apoptosis and detachment are mediated by TLR2. Finally, this can induce superficial erosion and thrombosis especially in VSMC‐rich plaques 170.

Pro‐inflammatory cytokines such as TNF‐α promote neutrophil recruitment 171. IL‐17A also exhibit stimulatory effects on neutrophils by generating a pro‐inflammatory neutrophil subset 172 and promoting neutrophil recruitment 173.

### Neutrophil granule components in atherosclerosis

In degranulation, activated neutrophils release large amounts of diverse cytotoxic and destructive factors capable to induce dramatic damage of vascular tissue. For example, neutrophil MPO contributes to ROS formation that in turn can be involved in oxidative modification of LDL and activation of endothelial cells thereby promoting a pro‐atherogenic environment 174. MPO also mediates irreversible protein modification *via* nitrosylation and the formation of 3‐chlorotyrosine and dityrosine crosslinks in the protein molecule 175, 176, 177. Jerke with co‐authors observed direct MPO transfer from neutrophils to endothelial cells mediated by β_2_‐integrin upon cell–cell contact followed by MPO‐mediated endothelial dysfunction 178. Granule‐derived proteases such as MMP‐1, MMP‐2, MMP‐9, neutrophil elastase and cathepsin participate in ECM degradation and therefore can contribute to vascular remodelling, fibrous cap thinning and atherothrombosis 179, 180, 181.

It should be stressed that neutrophils, monocytes and macrophage cooperate in induction and propagation of atherosclerotic inflammation. Neutrophils attract and guide inflammatory monocytes to plaques. Depletion of neutrophils results in decreased monocyte/macrophage counts within aortic lysates from mice 18. Neutrophils release a number of proteins such as azurocidin, cathepsin G, α‐defensins and LL37 (Cramp in mouse) that exhibit direct chemotactic activity on monocytes 182. Cramp/apoE‐deficient mice develop less advanced plaques with reduced monocyte/macrophage content. Cramp is deposited by neutrophils on the surface of inflamed endothelium. In this place, cathelicidins mediate adhesion of neutrophils and classical monocytes 183. The mechanism by which Cramp assists in the monocyte recruitment involves release of this peptide by extravasated neutrophils and reverse trafficking across the endothelium to the lumen where Cramp stimulates formyl‐peptide receptor 2–mediated β _1/2_ integrin activation and triggers monocyte recruitment 184. In humans, LL37 promotes recruitment of neutrophils, monocytes and T cells in a similar fashion employing formyl‐peptide receptor‐like 1 (FPRL1) 185.

Antimicrobial peptides α‐defensins exhibit pro‐atherogenic activity by enhancing monocyte recruitment, platelet activation and generation of foam cells 186. Azurocidin 1 (also known as cationic antimicrobial protein CAP37) stimulates monocyte recruitment through stimulation of surface expression of adhesion molecules (E‐selectin, ICAM1 and VCAM‐1) in endothelial cells 187, 188.

Lipocalin‐2 (also known as neutrophil gelatinase‐associated lipocalin; NGAL) is a small antimicrobial protein. In cardiovascular pathology, serum NGAL concentrations are elevated (particularly in subjects with carotid atherosclerosis and ischaemic cerebrovascular disease 189, 190, 191, 192 and serve as a marker of neutrophil activation and inflammation in preclinical atherosclerosis 193, 194. Furthermore, NGAL was shown to have a value as a prognostic marker for adverse cardiovascular events in patients with ST‐segment elevation myocardial infarction 195, acute myocardial infarction 196 and at‐risk subjects randomly selected from a general population 197, 198.

S100 calcium‐binding protein A9 (S100A9) may represent another potential mediator of neutrophil‐driven atherogenesis. This protein is constitutively expressed by neutrophils and other myeloid cells. S100A9 forms heterodimeric complex with S100A8 199. In the aorta, S100A9/apoE‐deficient mice had plaques of less size thereby suggesting for the pro‐atherogenic action of this protein 200. In patients with type 1 diabetes, serum levels of S100A9 were increased and correlated with higher neutrophil levels and coronary artery disease 82. The S100A9/8 complex promotes myelopoiesis and neutrophil migration as blockage of S100A9/8 results in reduced motility of neutrophils 201, 202. In infection, S100A9 is responsible for mobilization and recruitment of myeloid cells. However, in pathological conditions such diabetes or atherosclerosis, function of this protein may be compromised 203.

### NETs in atherosclerosis

NETs were detected in the luminal regions of human and murine lesions 204. Recently, Borissoff with co‐authors showed correlation between increased circulating NET levels and atherothrombosis 205. The presence of self‐DNA complexes with neutrophil granular proteins (that compose NETs) in the circulation activates plasmacytoid DCs (pDCs) and induces pro‐atherogenic pDC‐driven type I interferon (IFN) response 206. In ApoE‐deficient mice, pDCs were shown to aggravate atherosclerosis‐associated inflammation 207. Furthermore, Villanueva *et al*. 208 observed cytotoxic effects of NETs against ECs in inflammation. In ApoE‐deficient mice, Warnatsch *et al*. 209 showed that cholesterol crystals induce generation of NETs, which in turn activate Th17 cells and macrophage‐driven IL‐1β release. Platelet‐derived chemokines stimulate NET production 210. NETs were shown to stimulate local pro‐coagulant properties by thrombin generation *via* platelet‐dependent mechanisms 211. On the other hand, NETs could reciprocally enhance platelet activation and thereby promote atherothrombosis as demonstrated in a murine model of deep vein thrombosis 212.

## Conclusion

Impaired cholesterol efflux is crucially involved in ectopic fat accumulation in the arterial wall and the development of atherosclerotic disease. High‐blood cholesterol significantly promotes atherogenesis in obese and dislipidaemic people through several mechanisms, including induction of HSPC proliferation and mobilization from the bone marrow, and activation of preferential differentiation of HSPCs to myeloid precursors, which leads to the increase of pro‐inflammatory immune cells population 213. Although the overall beneficial effect of lipid‐lowering therapy towards the anti‐inflammation, cardioprotection and vasculoprotection is well established, it is difficult to estimate or predict the total outcome due to the multiplicity of molecular mechanisms and physiological functions targeted by statins and other lipid‐lowering agents.

Therapeutic approaches to exploit the anti‐atherogenic potential of HDL, for example with help of ApoA1 mimetic peptides or attempts to increase HDL levels in the circulation had promising effects in animals 214. However, in humans, clinical studies with the use of therapeutic agents designed to raise HDL failed to reach significant reduction of the cardiovascular risk 215, 216, 217.

Topics considered in this review may provide a more specific focus on the regulation of HSPC proliferation and differentiation through targeting reverse cholesterol transport pathways to prevent monocytosis/neutrophilia and reduce atherosclerotic inflammation. In experimental mouse models of atherosclerosis, activation of LXR‐dependent pathways in HSPCs was shown to inhibit their proliferation and pathological myelopoiesis, suppress foam cell formation, an attenuate inflammation and plaque progression. Due to significant curative actions shown by LXR agonists in experimental models of atherogenesis, these agents have a good therapeutic potential to be used for targeting cholesterol efflux mechanisms.

## Conflict of interest

The authors declare no conflict of interest.
